# GScluster: network-weighted gene-set clustering analysis

**DOI:** 10.1186/s12864-019-5738-6

**Published:** 2019-05-09

**Authors:** Sora Yoon, Jinhwan Kim, Seon-Kyu Kim, Bukyung Baik, Sang-Mun Chi, Seon-Young Kim, Dougu Nam

**Affiliations:** 1 School of Life Sciences, Ulsan National Institute of Science and Technology, Ulsan, Republic of Korea; 2 Epigenomics Research Center, Korea Research Institute of Bioscience and Biotechnology, Daejeon, South Korea; 3 Genome Structure Research Center, Korea Research Institute of Bioscience and Biotechnology, Daejeon, South Korea; 4 School of Computer Science and Engineering, Kyungsung University, Busan, Republic of Korea; 5 Department of Functional Genomics, University of Science and Technology (UST), Daejeon, 34141 Republic of Korea; 6 Genome Editing Research Center, Personalized Genomic Medicine Research Center, Korea Research Institute of Bioscience and Biotechnology (KRIBB), Daejeon, 34141 Republic of Korea; 7 Department of Mathematical Sciences, Ulsan National Institute of Science and Technology, Ulsan, Republic of Korea

**Keywords:** Gene-set clustering, Gene-set analysis, Protein-protein interaction, Network

## Abstract

**Background:**

Gene-set analysis (GSA) has been commonly used to identify significantly altered pathways or functions from omics data. However, GSA often yields a long list of gene-sets, necessitating efficient post-processing for improved interpretation. Existing methods cluster the gene-sets based on the extent of their overlap to summarize the GSA results without considering interactions between gene-sets.

**Results:**

Here, we presented a novel network-weighted gene-set clustering that incorporates both the gene-set overlap and protein-protein interaction (PPI) networks. Three examples were demonstrated for microarray gene expression, GWAS summary, and RNA-sequencing data to which different GSA methods were applied. These examples as well as a global analysis show that the proposed method increases PPI densities and functional relevance of the resulting clusters. Additionally, distinct properties of gene-set distance measures were compared. The methods are implemented as an R/Shiny package GScluster that provides gene-set clustering and diverse functions for visualization of gene-sets and PPI networks.

**Conclusions:**

Network-weighted gene-set clustering provides functionally more relevant gene-set clusters and related network analysis.

**Electronic supplementary material:**

The online version of this article (10.1186/s12864-019-5738-6) contains supplementary material, which is available to authorized users.

## Background

Gene-set analysis (GSA) covers a broad category of methods used to identify relevant biological pathways or functions from omics data such as microarray or high throughput sequencing data [1–4]. In many cases, GSA yields tens to hundreds of significant gene-sets without indicating how they interact with each other, rendering it difficult to identify core pathways or functional groups. Annotation databases such as Gene Ontology and KEGG [5, 6] partially address this issue by providing parent-offspring relations between annotation terms when used for GSA. Other gene-set collections obtained from independent and heterogeneous sources (e.g., gene signatures in MSigDB [7]) even lack such partial relations. Gene-set clustering, which helps identify the organization of gene-sets and their biological themes, has been used for improved interpretation of gene-sets. For example, DAVID web server uses Cohen’s kappa distance, and Enrichment map uses Meet/Min distance to cluster gene-sets into a number of subgroups [8–11]. However, these distance measures are only based on gene counts in each gene-set category (e.g., overlap between two gene-sets) and may not fully reflect the underlying biological relations such as protein-protein interactions (PPIs) between gene-sets.

Once significant gene-sets are identified, these GSA results can be further considered for a mechanistic study. PPI networks related to these gene-sets can provide useful information for this purpose. However, most GSA tools provide only the list of significant gene-sets [1] or their own networks [12, 13] without visualizing PPI networks between gene-sets. In this study, we propose to use a network-weighted distance for clustering gene-sets and present an R/Shiny package, GScluster (https://github.com/unistbig/GScluster), for clustering and network analysis of GSA results. The network-weighted clustering was better able to capture functionally relevant gene-set clusters compared with existing gene-count-based methods in simulated and real data analyses. GScluster accepts any GSA results from external programs if a list of gene-sets and their member genes (with or without gene-set scores) are provided.

A main goal of our analysis is to identify functionally relevant gene-set clusters from a long list of gene-sets; thus, the networks between genes (or proteins) in our analysis can be any kind of functional interaction such as gene co-expression, co-occurrence in the literature, evolutionary distance, physical contact, or their combinations, which were all simply denoted as PPI in this article. In GScluster, we adopted the STRING networks that integrated seven different functional interaction sources [14]. These networks also provide the widest coverage of species and genes among currently available network data (e.g., over 18,000 human genes). GScluster also provides analysis based on HIPPIE 2.0 networks [15] for human and customized network data.

To our knowledge, GScluster is the first attempt to incorporate both overlapping genes and PPI networks when clustering gene-sets. DAVID and Enrichment Map neither consider PPI networks during clustering gene-sets nor visualize PPI networks for the clustered gene-sets. Because both tools deploy different clustering strategies and DAVID only uses its own pathway gene-sets, a direct comparison between gene-set clustering results is not possible. Therefore, in our comparative analysis, we applied the same clustering strategy (fuzzy clustering) and compared the clustering results for three different distance measures: The Meet/Min distance of Enrichment Map (denoted as MM), Cohen’s kappa distance used in DAVID (denoted as KAPPA), and the PPI-weighted MM distance of GScluster (denoted as pMM). We also note that fuzzy clustering method allows a gene-set to belong to multiple clusters and excludes isolated gene-sets.

## Results

### GScluster R package and GSAseq web server

We present two independent software tools: GScluster (https://github.com/unistbig/GScluster) and GSAseq (http://gsaseq.appex.kr). GSAseq is a web server for gene-set enrichment analysis (GSEA) of RNA-sequencing (RNA-seq) and microarray gene expression data [7] and is described in Supplementary Material (Additional file 1) in detail. GScluster is an R/Shiny package for clustering and network analysis of gene-sets. We place a major emphasis on GScluster for its novelty and useful functions. GSEA and differential gene expression results obtained from GSAseq can be directly used for GScluster. Both software tools support analysis for ten species including human, mouse, rat, fly, zebra fish, rice, *A. thaliana*, worm, yeast, and *E. coli*. We note that GSA result from our own tool for GWAS summary data is also directly accepted by GScluster [16].

**Fig. 1:**
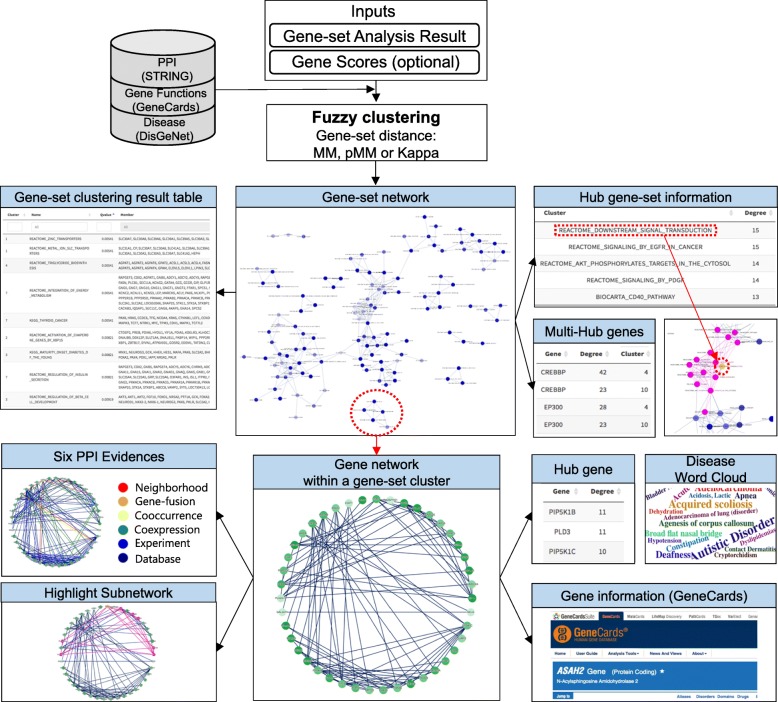
GScluster overview. Interactive network analysis is provided for both gene (protein) and gene-set networks

An overview of GScluster is shown in Fig. 1. The user can also use a GSA result (and gene list) obtained from other software tools for GScluster. The main input data should have the columns of gene-set name, member genes, gene-set score (e.g. FDR q-value), and optionally the direction of each gene-set indicating up- or down-regulation. GScluster additionally accepts data for gene scores (e.g. differentially expressed (DE) genes) for more focused analysis. GScluster implements the fuzzy clustering [8] for the three set-distance measures (pMM, MM, and KAPPA). GScluster is mainly built based on Shiny and Cytoscape related R package (cyjShiny); thus it provides interactive visualization of both gene-set and PPI networks. Networks are visualized after gene-set clustering is done. All the network figures and gene-set clustering result table are downloadable as SVG and CSV format, respectively. Detailed functions for network visualization are described in Supplementary Material (Additional file 1). GScluster clusters gene-sets and visualizes networks in seconds to minutes, depending on the size of input gene-set data (Fig. S1). The three example datasets (GSA results) analyzed in this article are available in the GScluster package.

### Network-weighted distance yields gene-set clusters with denser network connections

**Fig. 2:**
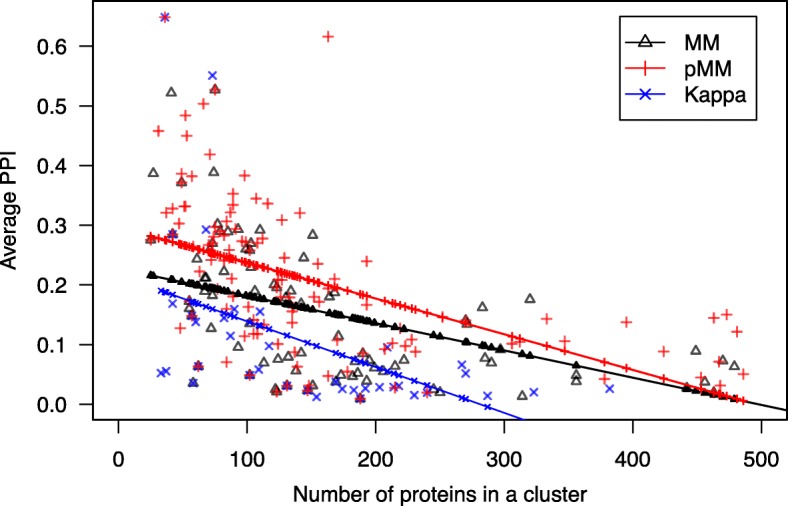
Comparison of average PPI scores within clusters generated using each of three distance scores. All of the MSigDB C2 pathways satisfying a set size criterion 10–200 were clustered using each of pMM, MM, and KAPPA distances. The average PPI scores (STRING) within each cluster were plotted for the numbers of proteins in clusters. For each method, the 1st order trend lines are represented (MM: black triangle, pMM: red cross. KAPPA: blue X)

Gene-set clustering aims at identifying groups of functionally close gene-sets that can be characterized by substantial overlap and dense PPIs between gene-sets. Here, we cluster the 3859 MSigDB C2 pathway sets [7, 17] with sizes 10–200 using pMM, MM, and KAPPA distances, respectively, and compare the PPI densities of resulting clusters. For each of the three distances, the same upper 0.154% threshold (MM ≤ 0.5, pMM ≤ 0.384, and KAPPA ≤ 0.727) was applied. Then, gene-set clusters with similar sizes (number of genes included) are compared between different methods. Because the sizes of resulting clusters are not exactly matched between methods, the trend lines between the cluster size and average PPI score (STRING edge scores are normalized to unit interval) in each cluster were compared (Fig. 2). Here, gene pairs with no PPI received zero scores in calculating the cluster averages, and a few outlier clusters with more than 1000 genes were excluded (2, 3, and 1 clusters for MM, pMM, and KAPPA, respectively). As expected, the average PPI scores tended to decrease as the cluster size increases for all the three methods. Indeed, the clusters obtained using pMM exhibited considerably increased average PPI scores than those observed using existing methods. For the clusters with less than 100 genes (39.8, 30.3, and 36.8% of pMM, MM, and KAPPA clusters, respectively), the average PPI score of pMM clusters (0.30) was 20 and 50% higher than those of MM (0.25) and KAPPA (0.20) clusters, respectively. For the clusters containing 100–200 genes (36.4, 41.6, and 31.6% of pMM, MM, and KAPPA clusters, respectively), even higher rates of increase were observed (average PPI scores using pMM: 0.17, MM: 0.12, and KAPPA: 0.05). These results illustrate the effects of incorporating PPI-networks in gene-set clustering, which can be controlled by the balancing factor α. Simulation tests were demonstrated in the next section to further compare the features of different distance measures.

### Gene-set clustering simulation

**Fig. 3:**
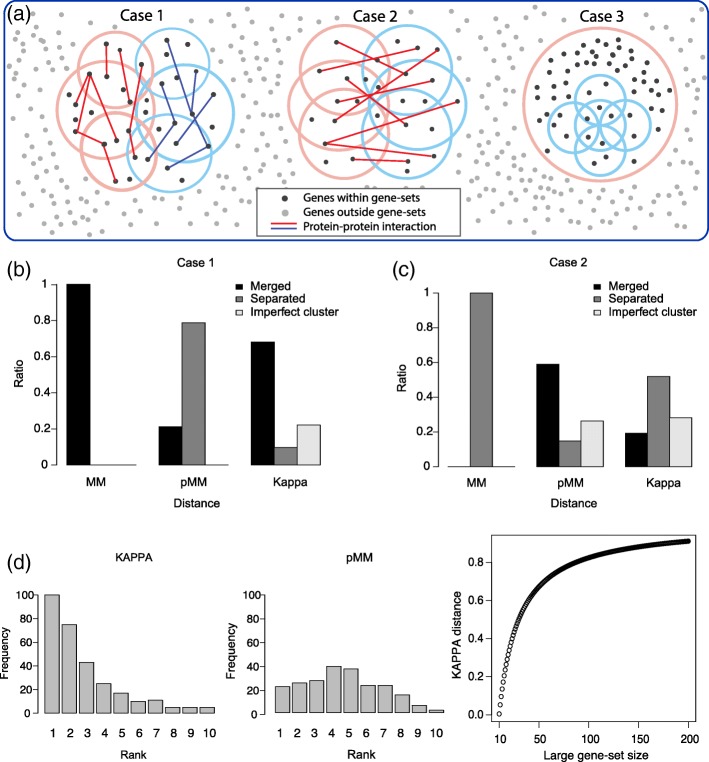
Simulation of gene-set clustering. **a** Three models for gene-set clusters. Dots represent genes and circles represent gene-sets. First model (Case 1) assumes that two subgroups (orange and sky-blue) are merged using MM distance but contain dense PPIs within each subgroup. Second model (Case 2) represents two subgroups having insufficient overlap to be merged using MM score, but containing dense PPIs across the subgroups. In the first and second models, only three gene-sets (instead of five) were represented in each subgroup to simplify the figures. In third model (Case 3), a large gene-set contains multiple small gene-sets having overlaps. Black and gray dots represent genes included in gene-set and background, respectively. Lines represent PPIs between genes. Clustering results for (**b**) Case 1 and (**c**) Case 2 are shown. Ratios in the y axes indicate the successful detection ratios. Results for Case 3 was not shown because all trials showed the same result for each distance measure (See the text). **d** Rank distributions of gene-set sizes of unclustered gene-sets in the imperfectly merged instances in Case 2. **e** A simulation for KAPPA where set A (*n* = 10) is contained in another set B (n = 10–200). KAPPA is strongly affected by the set-size

We designed three gene-set clusters as demonstrated in Fig. 3a to compare the effects of different gene-set distances on gene-set clustering. ‘n’ represents the number of genes in a gene-set. The properties of each cluster were described as follows:

1. **Case 1:** Ten gene-sets are largely classified into two functionally distinct subgroups (left and right parts), each composed of five gene-sets. The two subgroups share a substantial number of genes; however, PPIs are assigned only within each subgroup.
2. **Case 2:** Similar as Case 1 except that the two subgroups share fewer genes and PPIs are assigned only between the subgroups.
3. **Case 3:** A large gene-set (*n* = 200) includes five small gene-sets (*n* = 10–15) that overlap with each other.

Each case was simulated 500 times. The number of total genes was fixed as 10,000. In Case 1 and 2, each gene-set (*n* = 15–40) was sampled from either of two pools of genes (denoted pool 1 and 2, respectively), each with *n* = 60. These pools shared 20 genes. In Case 1, five gene-sets for the first subgroup were sampled from pool 1, and the other five for the second subgroup from pool 2. Each gene-set in a subgroup has a counterpart gene-set in the other subgroup and these gene-set pairs have 45–50% of common genes. In Case 2, the gene-set pairs had a lower proportion of overlap of (40–45%). PPIs with scores of 0.15–0.99 were assigned to 40% of gene (protein) pairs *within* each subgroup (Case 1), or *across* the subgroups (Case 2). PPI scores were randomly sampled from STRING networks and the resulting average PPI score for all gene pairs was 0.11. In Case 3, small gene-sets were sampled from a common pool of 50 genes to generate overlap among them. In all cases, the clustering cutoff of MM = 0.5 was used, and those for the other two distances were determined based on the corresponding percentile values in each dataset.

In Case 1, the two subgroups were merged into one 113 times out of 500 trials using MM. Among these, the two subgroups were mostly separated when pMM was used (89/113 times, 78.8%). In contrast, when KAPPA was used, the two subgroups were separated only 11 times (9.7%) and still merged 77 times (68.2%); in the remaining 25 cases (22.1%), ten gene-sets were imperfectly merged (Fig. 3b) which means some of the gene-sets were not clustered. In Case 2, the two groups were separated 156 times out of 500 trials using MM. Among them, pMM mostly merged the two subgroups (92/156 times, 59.0%) and separated them only 23/156 times (14.7%). In the remaining 41 cases (26.3%), the two subgroups were imperfectly merged because the missing gene-sets had relatively less number of PPIs with the clusters (average PPI score within merged gene-sets: 0.355; average PPI score between merged and not merged gene-sets: 0.249). KAPPA merged or separated the two groups 30 (19.2%) and 82 times (52.6%), respectively, and imperfect merging was observed 44 times (28.2%) (Fig. 3c). These simulation results demonstrate that pMM is capable of discriminating functionally distinct gene-set subgroups as represented by PPI networks. Small gene-sets were often missed from the merged cluster when KAPPA was used, whereas pMM did not show such a tendency (Fig. 3d).

Case 3 highlights the difference between KAPPA and MM/pMM distances. KAPPA excluded the large superset (*n* = 200) and detected only the cluster of five small gene-sets, whereas MM and pMM included the large superset into a single large cluster as well. Given two gene-sets, MM/pMM distances focused on the smaller gene-set and assumed their distance was zero if one gene-set was completely included by the other, irrespective of the size of the latter gene-set. By contrast, KAPPA tended to cluster gene-sets with similar sizes. To demonstrate the set-size dependence of KAPPA, a simple simulation was devised. Suppose a set A has ten members that are all contained in another set B. Then, KAPPA between A and B was measured for varying sizes of B (10–200). The size of B strongly affected the kappa distance (Fig. 3e), whereas MM distance was 0 irrespective of the size of B.

### Gene-set clustering/network analysis of real data

Complex diseases are caused by aberrant modulation of multiple pathways. Thus, gene-set analysis of a complex disease often yields a long list of significant pathways, making it difficult to identify core themes and overall relations among the pathways. Here, we demonstrate the network analysis of gene-sets using GScluster for three datasets of complex diseases: Colorectal cancer (CRC), type 2 diabetes (T2D), and acute myeloid leukemia (AML). These datasets have different data types (gene expression microarray, GWAS summary, and RNA-seq) and were analyzed using different GSA methods (GSEA, empirical self-contained GSA, and adaptive Gene Ontology (GO) analysis). In each example, clustering results based on pMM, MM, and KAPPA were compared, and PPI networks in gene-set clusters were analyzed. Only gene-sets with between 10 and 200 genes were analyzed.

#### GSEA of gene expression microarray data (colorectal cancer)

**Fig. 4:**
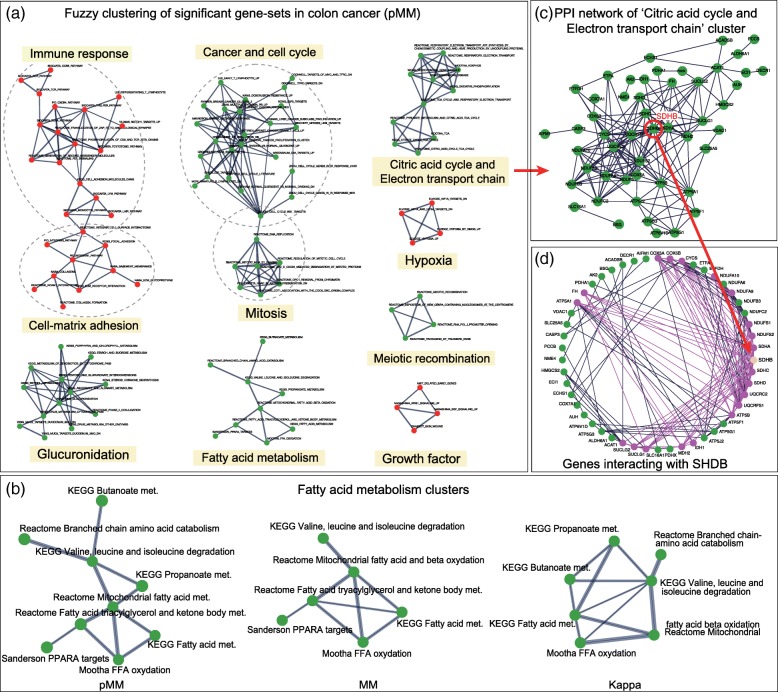
Gene-set network analysis of early-onset colorectal cancer data. **a** Gene-set networks/clusters obtained using pMM (GSEA FDR < 0.01). Pink and green nodes represent up- or down-regulation of gene-sets, respectively. Overlapping clusters were represented by dashed circles. The cluster labels were manually written by the authors. The sizes of node and gene-set name are adjustable on the web. **b** Gene-set clusters of fatty acid metabolism derived from pMM, MM, and KAPPA, respectively. In the pMM cluster, nodes bordered by orange and blue boxes indicate gene-sets that were not included in the MM and KAPPA cluster, respectively. **c** PPI network (score ≥ 0.5) of significant genes (FDR < 0.01) in cellular respiration cluster. SDHB was the hub (degree = 25). **d** PPI networks connected with SDHB in cellular respiration cluster. Met represents Metabolism

Microarray gene expression data (GSE4107) comprising 12 early onset CRC samples and 10 healthy controls were analyzed [18]. Differential expression analysis was performed using limma R package [19], and gene-permuting GSEA (gene score: log fold-change) was performed using GSAseq and MSigDB curated gene-sets (C2 category) [7]. The analysis results were then input into GScluster. In total, 484 enriched gene-sets (false discovery rate (FDR) < 0.01) and 1071 DE genes (FDR < 0.01) were used for network analysis. Figure 4a shows the gene-set clusters and networks generated using pMM. Each cluster contained gene-sets related to cancer such as cellular respiration (TCA cycle; electron transport chain pathways) [20, 21], fatty acid metabolism [22], immune response [23], cell cycle and apoptosis [24, 25], growth factor [26], and hypoxia [27, 28].

Fuzzy clustering of the 484 significant gene-sets generated 11, 10, and 14 clusters using MM, pMM, and KAPPA, respectively (Additional file 2: Supplementary Table S1). Overall, the three distances resulted in similar clusters; however, some clusters revealed distinctive features of each distance, as demonstrated in the simulation test. For example, ‘fatty acid metabolism’ cluster included six related terms using MM (e.g., KEGG fatty acid metabolism and Reactome mitochondrial fatty acid beta oxidation). pMM extended this cluster with three additional terms related to short chain fatty acids (KEGG propanoate metabolism and KEGG butanoate metabolism) and vascular fatty acid transport (Reactome branched chain amino acid catabolism). Their additions were ascribed to the PPI weights; the average PPI score between the six and the additional three pathways was 0.106 which was 7.54 times higher than that of background genes (0.014) as calculated from all the 484 significant gene-sets. Compared with the pMM results, KAPPA removed the largest pathway (Reactome fatty acid triacylglycerol and ketone body metabolism, *n* = 145) and its small subset (Sanderson PPARa targets, *n* = 15) possibly because of the set-size differences.

‘Immune response’ cluster showed a similar pattern. It contained 15 gene-sets related to lymphocyte activation using MM (e.g., Biocarta TCR pathway, Biocarta T-helper pathway, Reactome CXCR4 pathway). pMM included three additional relevant pathways (‘Biocarta T cytotoxic pathway’, ‘Lee differentiating T lymphocyte’, and ‘Vilimas Notch1 targets up’) in this cluster. The average PPI score between the 15 MM pathways and three additional pathways were 0.041 which was 2.93 times higher than that of the background. Among the 18 gene-sets in the pMM immune cluster, KAPPA was only able to cluster eight small immune response gene-sets (*n* = 11–25).

We then analyzed an extended list of 1147 gene-sets obtained from a larger threshold FDR < 0.1. Clustering using pMM yielded a large cluster related to ‘cell survival, proliferation, and differentiation’ (*N* = 67; N represents the number of gene-sets in a cluster, see in Additional file 1: Figure S2a ). In this cluster was found an important pathway in cancer, ‘Reactome activation of the AP1 family of transcription factors’. This pathway was the most highly connected (degree = 29) among the entire list of gene-sets in the pMM gene-set networks. This result is very relevant, because AP-1 is a well-known key transcriptional regulator for cell survival, proliferation, and differentiation in cancer [29, 30], and its activation pathway is connected to many related cell signaling pathways. In contrast, using MM or KAPPA, this gene-set was not detected as a hub and was connected to only a small number of gene-sets (MM: seven sets, KAPPA: three sets, see in Additional file 1: Figure S2b). We note that the gene-set clustering results for different distance measures have similar numbers of edges between gene-sets (pMM: 1242; MM: 1112; KAPPA: 1252) because the same percentile cutoff values were applied. Therefore, this example reveals a critical difference in the network structures with or without PPI weights.

GScluster enables to explore the PPI networks within each cluster, and easily identify the hub genes and their neighbors. For example, a cluster of ‘cellular respiration’ contained 11 gene-sets related to TCA cycle and oxidative phosphorylation. These gene-sets were down-regulated in colorectal cancer because of the Warburg effect that cancer cells exploit aerobic glycolysis rather than oxidative phosphorylation to produce energy [31]. The PPI network of this cluster contained 66 genes (PPI score ≥ 0.5). Among them, succinate dehydrogenase B (SDHB), participating in both citric acid cycle and respiratory chain [32], was the hub having connections with 25 genes. Deficiency of this gene increases cancer cell migration and invasion by activating the transforming growth factor (TGF) beta signaling pathway [33, 34]. Figure 4c, d represent the PPI networks of SDHB in this cluster.

#### Self-contained GSA of GWAS summary data (type 2 diabetes mellitus)

**Fig. 5:**
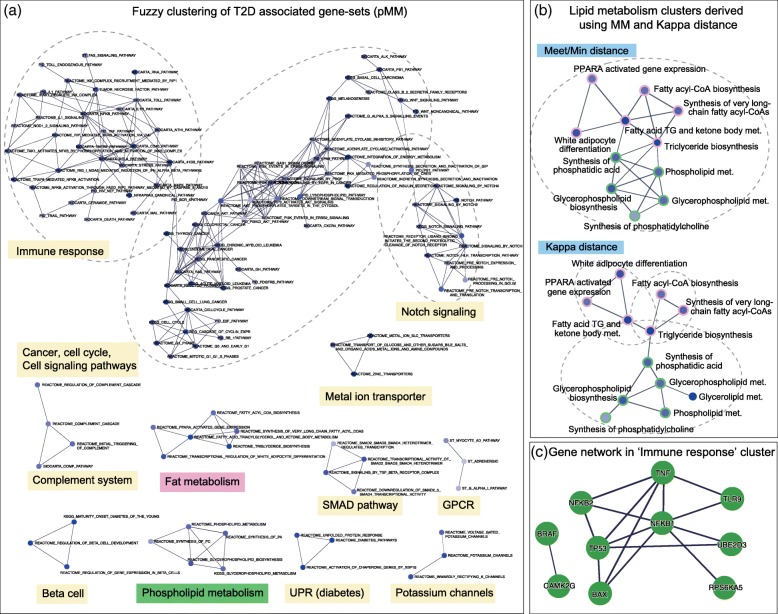
Gene-set network analysis of Type 2 Diabetes. **a** Gene-set networks/clusters obtained using pMM (sARTP FDR < 0.25). Overlapping clusters are represented using dashed circles. The cluster labels are manually written by the authors. UPR means unfolded protein response. **b** Clusters of lipid metabolism derived by MM (left) and KAPPA (right). Whereas pMM separated triglyceride (fat) and phospholipid metabolism pathways, MM and KAPPA distance combined them into one cluster. Also, KAPPA added another gene-set in the cluster. Green and pink border represent the gene-sets related to phospholipid and fat metabolism, respectively. TG means Triglyceride. **c** PPI network (PPI score ≥ 0.5) of significant genes (FDR < 0.01) in the immune cluster. NFKB1, NFKB2, TNF, and TP53 were fully connected to each other

Next, stage 1 GWAS summary statistic data provided from the DIAGRAM consortium were analyzed [35]. This dataset was obtained from a meta-analysis comparing genotypes of 12,171 patients with T2D and 56,862 controls collected from 12 GWAS European populations. For this dataset, a recently developed empirical self-contained GSA method, called sARTP was applied using ARTP2 R package [36]. In addition, gene *p*-values were calculated using VEGAS2 tool [37]. sARTP method detected 193 significant gene-sets out of 1264 MSigDB C2 canonical pathways (FDR < 0.25). Fuzzy clustering summarized these sets into 12, 10, and 16 clusters using pMM, MM, and KAPPA, respectively (Additional file 2: Supplementary Table S2). Many clusters included gene-sets that were closely related to T2D such as beta cell regulation [38], unfolded protein response [39], Notch/Wnt/PS1 signaling [40], cell cycle [41, 42], signal transduction [43, 44], cancer [45], voltage-gated potassium channel [46, 47], immune response [48], and lipid metabolism [49]. The gene-set networks generated using pMM are shown in Fig. 5a.

Some of the clusters clearly revealed specific features of each distance measure as observed in the simulation test. For example, ‘lipid metabolism’ cluster corresponded to Case 1 and 3 in the simulation. Among the MM clustering results, a cluster included two distinct functions, phospholipid and triglyceride related terms (*N* = 11). It was ascribed to the overlap of a triglyceride term (Reactome triglyceride biosynthesis) with three phospholipid terms (Reactome synthesis of PA, Reactome glycerophospholipid biosynthesis, and Reactome phospholipid metabolism) (MM = 0.48–0.5). The 14 overlapping genes were general synthesis related genes (e.g., AGPAT/GPAT family, GPAM, and GPD1) that are involved in both triglyceride and phospholipid biosynthesis [50]. In contrast, pMM yielded two separate clusters of ‘phospholipid’ (*N* = 5) and ‘triglyceride metabolism’ (*N* = 6). Such different cluster structures were clearly caused by the PPI distribution; the average PPI score between the two clusters was even lower than that of the background (0.016, odds ratio = 0.496), whereas the average PPI scores within each cluster were much higher (phospholipid: 0.21, odds ratio = 8.39; triglyceride: 0.099, odds ratio = 3.46). This separation of clusters is also biologically reasonable because they represent clearly distinct functions; phospholipids are structural constituent of cell membranes, whereas triglycerides are used for energy storage [50].

Figure 5c shows the PPI network of ‘innate immune response’ cluster containing 31 gene-sets. It exhibited dense connections among ten well-known immune related genes (gene *p*-value < 0.01). In particular, four hub genes (NFKB1, NFKB2, TNF, and TP53) were completely connected to each other. The roles of these genes in T2D have been well-studied. Expressions of these pro-inflammatory genes are usually elevated in T2D. These are activated under obesity or high-fat diet conditions and cause an inflammatory response that leads to insulin resistance [51–53]. Besides, TP53 was also detected as a hub in another cluster related to cancer/cell cycle and cell signaling pathways. This cluster included many gene-sets altered in both cancer and diabetes such as cell cycle, Akt pathway, and MAPK pathway. It was reported that a dysregulated isoform of TP53 (Δ40p53) causes cell cycle arrest in beta cells and insulin resistance [54].

#### GO analysis of RNA-seq data (acute myeloid lymphoma)

Lastly, GO analysis was performed for RNA-seq data composed of induced pluripotent stem cells derived from three patients with AML and three healthy controls [55]. In total, 5307 DE genes were identified out of 21,441 genes (FDR < 0.01 and two or larger fold change) using DESeq2 [56]. GO analysis was performed for these genes using GOseq R package [57]. GOseq is specifically designed for GO analysis of RNA-seq data to address the read count bias (or gene length bias) in DE analysis of RNA-seq data [58, 59]. Among 5136 MSigDB C5 gene-sets, GOseq yielded 86 significant gene-sets (FDR < 0.01).

**Fig. 6:**
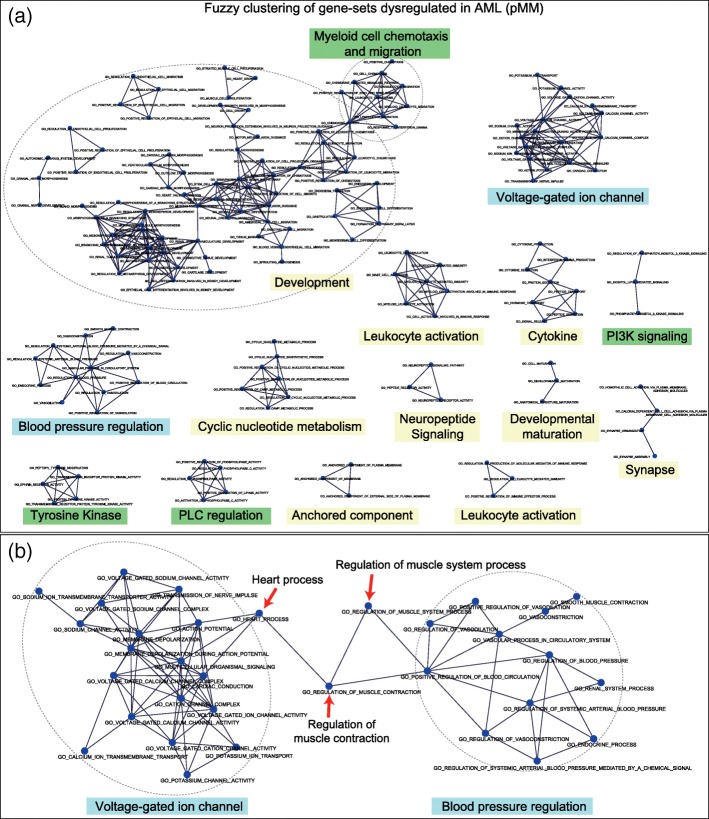
Gene-set network analysis of acute myeloid leukemia. **a** Gene-set networks/clusters obtained using pMM (GOseq FDR < 0.01). In this case, the nodes are colored in blue because GOseq results do not give the information of up- or down-regulation. The labels of clusters were manually added by the author. **b** An example KAPPA gene-set cluster. This cluster combined ‘voltage-gated ion channel’ and ‘blood pressure regulation’ clusters with connecting gene-sets (denoted by arrows)

Fuzzy clustering of these gene-sets yielded 15, 11, and 19 clusters using pMM, MM, and KAPPA, respectively (Additional file 2: Supplementary Table S3). These clusters summarized the characteristic pathways of AML, such as immune response [60, 61], synapse [62], anchored component of membrane (e.g., CD48, CD56) [63, 64], neuropeptide [65, 66], tyrosine kinase [67], developmental [68, 69], blood pressure regulation [70, 71], cyclic nucleotide [72, 73], voltage-gated ion channels [74, 75] and phospholipase C [76] (Fig. 6a).

Among the eleven clusters produced using MM, only one was different from those generated using pMM. It contained 94 gene-sets and the majority (77%) of them were related to the development of kidney, cartilage, cardiovascular system, and nervous system. Other sets were related to leukocyte chemotaxis and migration (LCM), protein tyrosine kinase (TK), phospholipase C (PC), and phosphatidylinositol (PI) regulation. When pMM was applied, these distinct sets were separated from the large cluster. The average PPI within each separated cluster was much higher than that of combined one (Average PPI: combined = 0.036, LCM = 0.073, TK = 0.113, PC = 0.132, PI = 0.149).

Clusters produced using KAPPA were quite different from those obtained using MM and pMM. For example, MM and pMM produced two distinct clusters of blood pressure regulation (*N* = 12) and voltage-gated ion channel (*N* = 18). Using KAPPA these clusters were combined into one with additional three gene-sets which worked as ‘mediators’ between the two clusters (Fig. 6b). The gene-set ‘heart process’ (*n* = 85) had no connection with the gene-sets in the voltage-gated ion channel cluster using MM or pMM. However, KAPPA connected it with three sets in this cluster (‘multicellular organismal signaling’ (*n* = 123), ‘cardiac conduction’ (*n* = 82) and ‘action potential’ (*n* = 94)) because of the similar gene-set sizes. Also, it was connected to ‘regulation of muscle contraction’ (*n* = 147) which was linked to a gene-set in the blood pressure cluster (‘positive regulation of blood circulation’ (*n* = 93)) only by KAPPA. In the large combined cluster, a small pathway ‘vasodilation’ (*n* = 26) was removed because it lost the link with its superset ‘vascular process in circulatory system’ (*n* = 163) using KAPPA because of the size difference.

## Discussion

Gene-set analysis often yields a long list of gene-sets. DAVID [8] or Enrichment Map [11] cluster those gene-sets to summarize the results and identify core themes regarding the phenotype of interest. However, these methods only consider ‘overlap’ based distances in clustering gene-sets, whereas functionally related gene-sets usually share a number of PPIs as well as some genes.

In the present study, we introduced a PPI-weighted gene-set distance (pMM) that incorporates both the overlapping genes and PPIs between two gene-sets. pMM was compared with existing distance measures, Meet/Min (MM) and kappa distance, in clustering a large collection of gene-sets (MSigDB C2), where pMM clusters, as expected, exhibited systematically higher PPI densities than those obtained using MM or KAPPA distances. pMM enabled to capture biologically more meaningful clusters as shown in three analysis examples. We also presented GScluster tool for clustering and network analysis of gene-sets. It accepts any kind of GSA results and helps to identify core biological themes from a long list of gene-sets.

Additionally, the unique properties of each distance measure were demonstrated from simulation and real data analysis. In particular, kappa distance used in DAVID was highly sensitive to gene-set size difference and tended to cluster gene-sets of similar sizes. This property of kappa distance should be taken into account when clustering the hierarchically organized gene-sets in GO and KEGG, because kappa distance may not cluster a large general pathway and its small sub-pathways.

In the colon cancer example, only the pMM-based clustering identified the well-known oncogenic complex, AP1 family pathway as hub gene-set. In the T2D example, two unrelated pathway groups (phospholipid and triglyceride synthesis) were clustered into one when the overlap-based distance (MM) was used, because they shared general synthesis-related genes. However, these two pathways have distinct functions and should not be taken together just because they share some non-specific genes. pMM reflected the relatively dense PPI scores within each group and successfully separated them into two distinct pathway groups.

These examples as well as the simulation results indicate that PPIs should be taken into account for gene-set clustering and network analysis. Whereas the default network weighting of α = 1 worked well for STRING networks in all the three examples in this paper, this factor could be reduced if the network data are of low quality or less reliable. By incorporating PPI networks, GScluster provided functionally more relevant gene-set clusters as well as corresponding PPI networks. Because gene-set clusters can be regarded as ‘extended’ pathways, PPIs in each cluster can provide useful insights for further study. In particular, GScluster showed that some proteins are hubs in multiple clusters, suggesting their multifaceted roles in diseases.

In clustering gene-sets, we implemented fuzzy clustering in GScluster package. This method has several advantages in summarizing a long list of gene-sets. First, it generates compact type clusters. Spectral clustering [77], for example, focuses more on the connectivity between nodes; thus, some pairs of nodes in a cluster can have very large distances. Second, the number of clusters is adaptively determined from the gene-set distance cutoff. Spectral clustering and *k*-medoids require a predetermined number of clusters which is not known to the user. Lastly, fuzzy clustering allows a gene-set to belong to two or more clusters. This flexibility is important because some gene-sets have important roles in multiple pathways. In contrast, many other clustering methods simply partition the gene-sets.

In addition to gene-set clustering, we expect that the PPI-weighted set distance (pMM) can also be used to design an enrichment analysis method (or GO analysis). Enrichment analysis typically evaluates enrichment of a test gene-set (e.g., DE genes from an experiment) in each pre-defined gene-set based on their overlap, whereas recent ‘network enrichment’ analysis methods consider enrichment of networks between gene-sets. pMM suggests a way to combine overlap and networks in enrichment analysis.

## Conclusions

Gene-set clustering has been widely used to summarized a long list of gene-sets. Here, we demonstrated that our PPI-network weighted gene-set distance yields biologically more relevant gene-set clusters by simulated and real data analysis. We also provided GScluster R/Shiny package for PPI-weighted gene-set clustering and network visualization.

## Methods

### Gene-set clustering and distance measures

The distance matrix between gene-sets are calculated using Meet/Min, PPI-weighted Meet/Min or Cohen’s kappa distance, and the fuzzy clustering algorithm used in DAVID was implemented in GScluster [8]. The distance measures used are described as follows:

1. Meet/Min distance (MM) for two gene-sets A and B:
  $$ \mathrm{MM}\left(\mathrm{A},\mathrm{B}\right)=1-\frac{\left|A\bigcap B\right|}{\min \left(\left|A\right|,\left|B\right|\right)} $$
  where |*A*| is size of A.
2. PPI-weighted Meet/Min (pMM) distance: For two gene-sets A and B,
  $$ \mathrm{pMM}\left(\mathrm{A}\to \mathrm{B}\right)=1-\frac{\left|A\bigcap B\right|}{\min \left(\left|A\right|,\left|B\right|\right)} $$
  1 $$ -\frac{\alpha }{\min \left(\left|A\right|,\left|B\right|\right)}\sum \limits_{x\in A-B}\frac{w{\sum}_{y\in A\cap B}P\left(x,y\right)+{\sum}_{y\in B-A}P\left(x,y\right)}{\max (P)\bullet \left(w\left|A\cap B\right|+\left|B-A\right|\right)} $$
  where *P* is the PPI score matrix, *P*(x, y) is the PPI score of two genes x and y, *α* ∈ [0, 1] is the balancing factor (default *α* = 1) used to control the network weighting based on quality or importance of the network data, and $$ \mathrm{w}=\left\{\begin{array}{c}\frac{\mid A\mid }{\left|A\right|+\mid B\mid }, if\ \left|A\right|\le \mid B\mid \\ {}\frac{\mid B\mid }{\left|A\right|+\mid B\mid }, otherwise\ \end{array}\right. $$; and pMM(B → A) is symmetrically defined. Then, the distance between A and B is defined as
  $$ \mathrm{pMM}\left(\mathrm{A},\mathrm{B}\right)=\min \left(\mathrm{pMM}\left(\mathrm{A}\to \mathrm{B}\right),\mathrm{pMM}\left(\mathrm{B}\to \mathrm{A}\right)\right). $$

**Fig. 7:**
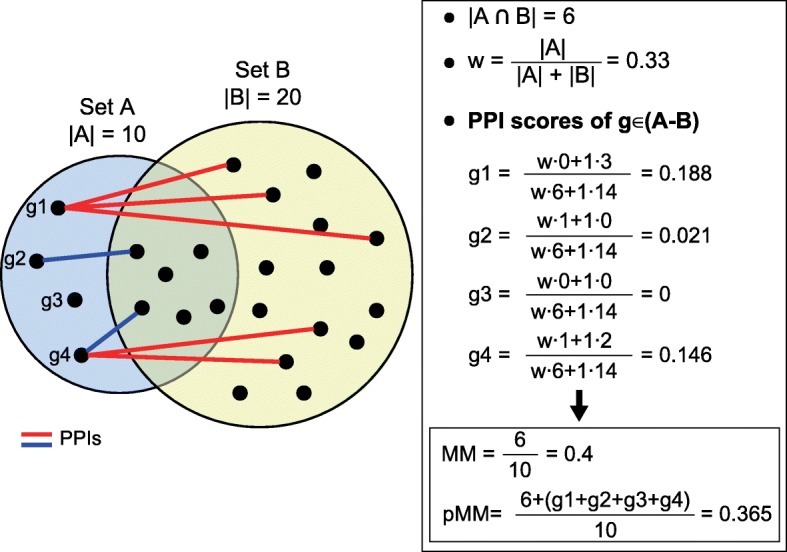
PPI-weighted gene-set distance. Two gene-sets A and B contain 10 and 20 genes, respectively, and share six genes. Red lines indicate PPIs between A-B and B-A, and blue lines, PPIs between A-B and A ∩ B. Here, all the PPI scores are simply assumed to be 1. Right table shows how to calculate pMM distance, and compares with MM distance value

The last term in (1) represents the overall PPI score between genes x ∈ A − B and y ∈ B. This term is designed as follows: if x ∈ A − B is fully connected with all the members in B with the maximum PPI score, this gene is regarded as one more overlap between A and B from the perspective of MM score. If y ∈ A ∩ B, the interaction score is down-weighted by *w* because the interaction in this case can be ambiguously regarded as that either within A or between A and B. This weight is determined by the probability that y belongs to the opposite set. For example, if |*A*| ≤  ∣ *B*∣, y is assumed to more likely belong to A; therefore the probability of y to belong to B becomes $\frac{1/\mid B\mid }{1/\left|A\right|+1/\mid B\mid }=\frac{\mid A\mid }{\left|A\right|+\mid B\mid }$. Although the PPI weighting has substantial effects on gene-set clustering, it also implies that effects of ‘hub’ genes with many strong connections with the opposite set are reasonably controlled. In general, pMM is less than or equal to MM, and they have the same value if there are no PPIs between two gene-sets. Because gene-set cluster structures are determined by the relative distances between gene-sets, some clusters can be dramatically changed by incorporating the PPI-weighted distance. Figure 7 illustrates how to calculate pMM between two gene-sets.

(3) kappa distance (KAPPA): Cohen’s kappa distance considers the background genes (*A* ∪ *B*)^*C*^ as well and is defined as:

$$ KAPPA\left(A,B\right)=1-\frac{O-E}{1-E} $$

where $\mathrm{O}=\frac{\left|A\cap B\right|+\mid {\left(A\cup B\right)}^c\mid }{\mid U\mid }$ and $\mathrm{E}=\frac{\left|A\right|\bullet \left|B\right|+\mid {A}^c\mid \bullet \mid {B}^c\mid }{{\left|U\right|}^2}$ are the observed and expected agreement rates of two gene-sets, respectively, and U is the set of all genes.

## Additional files

Additional file 1: Supplementary Material. This includes descriptions of GSAseq web server, gene-set collection method, network visualization and runtime of GScluster, and Supplementary Figure S2. (DOCX 1970 kb)

Additional file 2: **Table S1**, **Table S2**, and **Table S3.** Supplementary tables. Gene-set clustering results of the colon cancer, type II diabetes, and AML examples using three different distance measures. (XLSX 139 kb)

## Abbreviations

DE: Differentially expressed
FDR: False discovery rate
GO: Gene Ontology
GSA: Gene-set analysis
GSEA: Gene-set enrichment analysis
KAPPA: Cohen’s kappa distance
KEGG: Kyoto Encyclopedia of Genes and Genomes
MM distance: Meet/Min distance
pMM: PPI-weighted MM distance
PPI: Protein-protein interaction
