# Supplementary material for: GScluster: network-weighted gene-set clustering analysis
Source: BMC Genomics. 2019 May 9;20:352. doi: 10.1186/s12864-019-5738-6 (PMC6507172; doi:10.1186/s12864-019-5738-6)
Supplement: Supplementary file 1 — Supplementary Material. This includes descriptions of GSAseq web server, gene-set collection method, network visualization and runtime of GScluster, and Supplementary Figure S2. (DOCX 1970 kb) [file 12864_2019_5738_MOESM1_ESM.docx]

**Supplementary Material**

**GSAseq web server**

GSEA is one of the most widely used GSA methods for gene expression data [1]. We implemented the GSEA algorithm in C++ for a fast computation. RNA-seq count data with two sample groups are acceptable and are normalized by TMM [2] or DESeq median method [3]; however, already normalized read counts or normalized microarray data are also acceptable. The gene scores were calculated using signal-to-noise ratio (SNR), logarithmic fold-change, or zero-centered ranksum score [4]. Both gene and sample-permuting GSEA methods are provided. The absolute GSEA filtering method which is useful for reducing false positives in the gene-permuting method is also implemented. See the user’s manual or our previous paper [5] for a detailed description of the algorithms. It provides various collections of gene-sets as described below. Differential expression (DE) analysis of individual genes for RNA-seq/microarray are also performed using DESeq2 [6], edgeR [7], or limma [8] packages. Resulting significant genes can be used for more focused PPI network analysis.

**Gene set collection in GSAseq**

1. **Gene Ontology:** Gene ontologies (biological pathway, cellular component and molecular function) of nine species including human, mouse, rat, fruit fly, yeast, worm, zebrafish, rice and Arabidopsis thaliana were retrieved from biomart [9]. Gene ontologies of E.coli K12 strain was retrieved from Bioconductor package ‘ecoliK12.db0’ [10].
2. **KEGG:** KEGG gene sets for all nine species except rice were downloaded from KEGG FTP sites http://www.kegg.jp/kegg/download/
3. **TFBS:** To construct the gene set of transcription factor binding sites (TFBS) of human and mouse, TF binding domains obtained by **HT-selex** experiment of both species were retrieved from the Supplemental Table 2 of the paper by Jolma and colleagues [11]. The promoter sequence of each gene was set as -2kb to +2kb from the TSS (reference genome: GRCh38 for human and mm10 for mouse). TF target were searched by matching each TF binding domain and gene promoter using Biostrings package in R [12]. The TF binding domain for rat was obtained from JASPAR database [13] (reference genome: rn4).
4. **Chromosome:** The information of each gene’s location in the chromosome for human, mouse, rat and yeast was retrieved using the Bioconductor package ‘AnnotationDbi’ [14].
5. **MSigDB C1 (cytogenetic band), C2 (canonical pathway) and C5 (Gene ontology):** Downloaded from MSigDB (http://software.broadinstitute.org/gsea/msigdb/) [1].

**Implementation of GSAseq web service**

GSAseq web service implements JAVA and JAVA-based frameworks. To provide a user-friendly and an interactive interface, the Google web toolkit (GWT, ver. 2.6.1) and GWT extended (GXT, ver.3.1.1) frameworks were used. Data exchange between clients and the web server is controlled by a GWT remote procedure call. The normalization methods (TMM and DESeq) were implemented using the Bioconductor R packages edgeR and DESeq, respectively. The gene statistics and GSEA algorithm was implanted by C++ language environment (gcc ver. 4.8.3). To handle the multiple time-consuming biob concurrently, the Quartz framework was used (ver.2.1.6). To handle user analysis history, the MySQL database server was used (ver. 5.5.11).

**Extension of GSEA applications**

GSEA module can also analyze already normalized read counts or microarray data by simply choosing 'Already normalized' in the normalization option. Species besides the nine that are currently supported can also be analyzed by uploading corresponding gene-sets. A user's manual is available from the GSAseq web site (http://gsaseq.appex.kr). GSAseq supports three gene name systems for the input data: gene symbol, Ensembl and Entrez, but another gene name system can be also used if the corresponding gene-sets are uploaded.

**GScluster: Gene-set and gene network visualization**

GScluster provides both gene-set and gene (or PPI) network visualization. In gene-set network, the color intensity of each node represents the significance of the corresponding gene-set. The gene-set clustering result can be regenerated by applying different distance (MM, pMM or KAPPA; default = pMM), gene-set distance cutoff, minimum seed size of fuzzy clustering (default = 3), and the network weighting factor 𝒂 (default = 1). The default gene-set distances were set as the same percentile values corresponding to MM = 0.5. The selected gene-set cluster can be highlighted and corresponding PPI networks are visualized. Here, only the nodes with at least one PPI and edges larger than the threshold (default 0.7) are visualized. In PPI networks, the line width represents the STRING combined PPI scores [15, 16]. For simplicity, PPI scores of STRING that range from zero to 1,000 are rescaled to unit interval [0,1]. GScluster also visualizes six different types of PPI networks from STRING database (Neighborhood, Gene Fusion, Co-occurrence, Co-expression, Experiments and Database). Each gene node is hyperlinked to the GeneCards site where detailed gene functions are described [17]. From PPI networks, the wordcloud is generated based on the disease-gene relation information provided by DisGeNet [18]. For both gene-set and gene networks, the user can zoom in and out of the graphs, modify the size of nodes and text labels, and download the graph as .SVG format. In addition, both top five hub gene-sets and genes as well as the hub genes observed in multiple clusters are provided.

**GScluster runtime**

CPU times taken for clustering and network plots were measured by increasing the input gene-set size. It took only 23 seconds for 200 gene-sets, and the runtime was linearly increased by approximately 40 seconds for every 100 additional gene-sets. (CPU: Intel® Core™ i5-8265U processor, RAM 8G)

**Figure S1.** Runtime of GScluster.


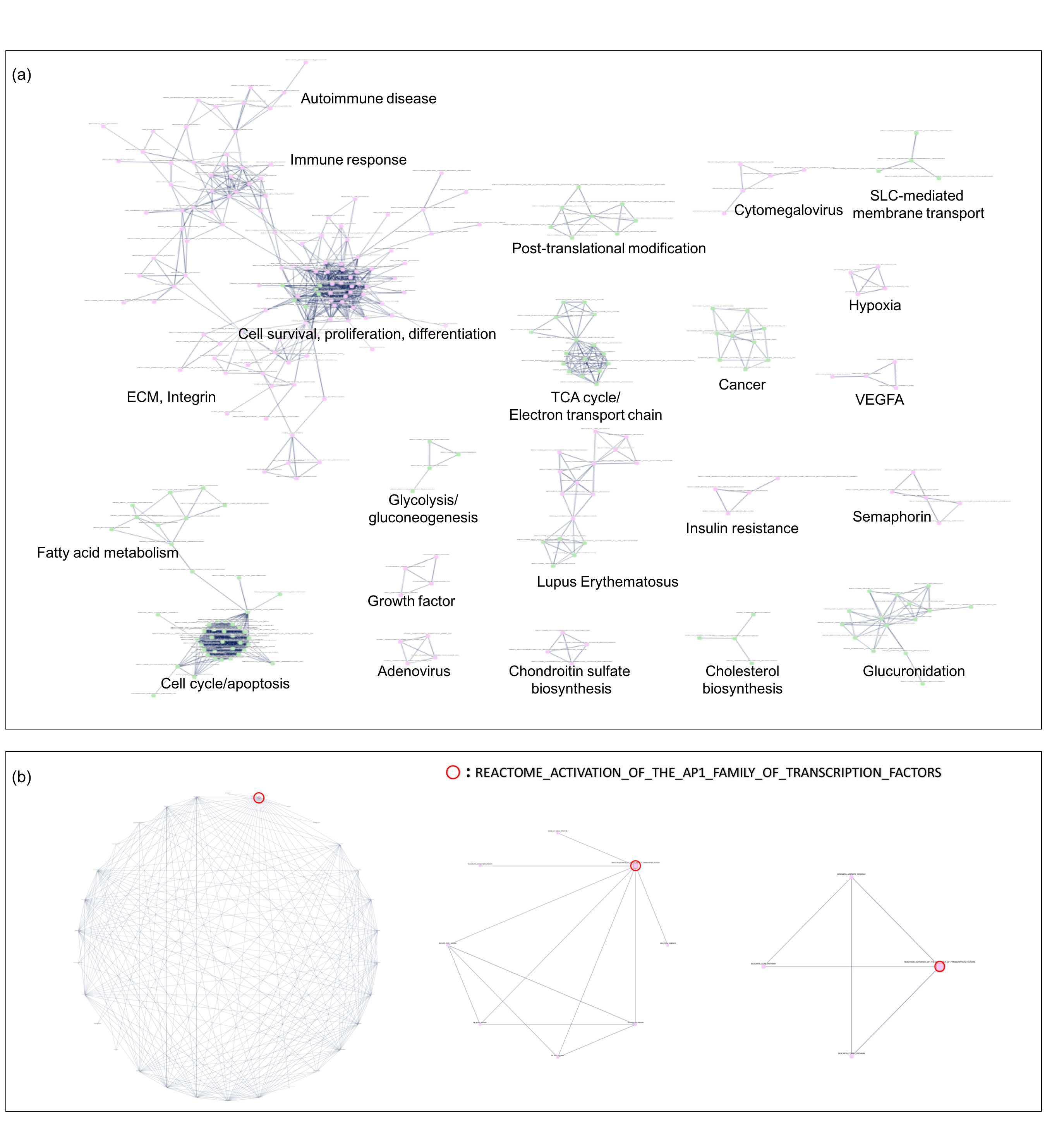


**Figure S2**. Gene-set network analysis of early-onset colorectal cancer data using extended list of gene-sets (GSEA FDR < 0.1). (a) Fuzzy-clustered gene-set networks using pMM. Nodes colored by pink and green represented up- and down-regulation of gene-sets, respectively. (b) Networks of gene-sets connected to ‘Reactome activation of the AP1 family of transcription factors’ (AP1 pathway) using pMM, MM, and KAPPA, respectively. AP1 pathway is marked by a red circle.

**Supplementary References**

1. Subramanian A, Tamayo P, Mootha VK, Mukherjee S, Ebert BL, Gillette MA, Paulovich A, Pomeroy SL, Golub TR, Lander ES *et al*: **Gene set enrichment analysis: a knowledge-based approach for interpreting genome-wide expression profiles**. *Proc Natl Acad Sci U S A* 2005, **102**(43):15545-15550.

2. Robinson MD, Oshlack A: **A scaling normalization method for differential expression analysis of RNA-seq data**. *Genome Biol* 2010, **11**(3):R25.

3. Anders S, Huber W: **Differential expression analysis for sequence count data**. *Genome Biol* 2010, **11**(10):R106.

4. Li J, Tibshirani R: **Finding consistent patterns: a nonparametric approach for identifying differential expression in RNA-Seq data**. *Stat Methods Med Res* 2013, **22**(5):519-536.

5. Yoon S, Kim SY, Nam D: **Improving Gene-Set Enrichment Analysis of RNA-Seq Data with Small Replicates**. *PLoS One* 2016, **11**(11):e0165919.

6. Love MI, Huber W, Anders S: **Moderated estimation of fold change and dispersion for RNA-seq data with DESeq2**. *Genome Biol* 2014, **15**(12):550.

7. Robinson MD, McCarthy DJ, Smyth GK: **edgeR: a Bioconductor package for differential expression analysis of digital gene expression data**. *Bioinformatics* 2010, **26**(1):139-140.

8. Ritchie ME, Phipson B, Wu D, Hu Y, Law CW, Shi W, Smyth GK: **limma powers differential expression analyses for RNA-sequencing and microarray studies**. *Nucleic Acids Res* 2015, **43**(7):e47.

9. Smedley D, Haider S, Durinck S, Pandini L, Provero P, Allen J, Arnaiz O, Awedh MH, Baldock R, Barbiera G *et al*: **The BioMart community portal: an innovative alternative to large, centralized data repositories**. *Nucleic Acids Res* 2015, **43**(W1):W589-598.

10. Pages MCaH: **ecoliK12.db0: Base Level Annotation databases for E coli K12 Strain**.

11. Jolma A, Yan J, Whitington T, Toivonen J, Nitta KR, Rastas P, Morgunova E, Enge M, Taipale M, Wei G *et al*: **DNA-binding specificities of human transcription factors**. *Cell* 2013, **152**(1-2):327-339.

12. H. Pages PAaRG, S. DebRoy: **Biostrings: String objects representing biological sequences, and matching**

**algorithms**.

13. Mathelier A, Zhao X, Zhang AW, Parcy F, Worsley-Hunt R, Arenillas DJ, Buchman S, Chen CY, Chou A, Ienasescu H *et al*: **JASPAR 2014: an extensively expanded and updated open-access database of transcription factor binding profiles**. *Nucleic Acids Res* 2014, **42**(Database issue):D142-147.

14. Herve Pages MC, Seth Falcon and Nianhua Li: **AnnotationDbi: Annotation Database Interface**.

15. Szklarczyk D, Morris JH, Cook H, Kuhn M, Wyder S, Simonovic M, Santos A, Doncheva NT, Roth A, Bork P *et al*: **The STRING database in 2017: quality-controlled protein-protein association networks, made broadly accessible**. *Nucleic Acids Research* 2017, **45**(D1):D362-D368.

16. Snel B, Lehmann G, Bork P, Huynen MA: **STRING: a web-server to retrieve and display the repeatedly occurring neighbourhood of a gene**. *Nucleic Acids Res* 2000, **28**(18):3442-3444.

17. Safran M, Dalah I, Alexander J, Rosen N, Iny Stein T, Shmoish M, Nativ N, Bahir I, Doniger T, Krug H *et al*: **GeneCards Version 3: the human gene integrator**. *Database (Oxford)* 2010, **2010**:baq020.

18. Goh KI, Choi IG: **Exploring the human diseasome: the human disease network**. *Brief Funct Genomics* 2012, **11**(6):533-542.
